# Statistical Genetic Approaches to Investigate Genotype-by-Environment Interaction: Review and Novel Extension of Models

**DOI:** 10.3390/genes15050547

**Published:** 2024-04-25

**Authors:** Vincent P. Diego, Eron G. Manusov, Marcio Almeida, Sandra Laston, David Ortiz, John Blangero, Sarah Williams-Blangero

**Affiliations:** 1 Department of Human Genetics, School of Medicine, University of Texas Rio Grande Valley, Brownsville, TX 78520, USA; eron.manusov@utrgv.edu (E.G.M.); marcio.almeida@utrgv.edu (M.A.); sandra.laston@utrgv.edu (S.L.); john.blangero@utrgv.edu (J.B.); sarah.williams-blangero@utrgv.edu (S.W.-B.); 2 South Texas Diabetes and Obesity Institute, School of Medicine, University of Texas Rio Grande Valley, Brownsville, TX 78520, USA; 3 Department of Family Medicine, School of Medicine, University of Texas Rio Grande Valley, Brownsville, TX 78520, USA; david.ortiz04@utrgv.edu

**Keywords:** polygenic model, heritability, genotype-by-environment interaction, depression, social determinants of health

## Abstract

Statistical genetic models of genotype-by-environment (G×E) interaction can be divided into two general classes, one on G×E interaction in response to dichotomous environments (e.g., sex, disease-affection status, or presence/absence of an exposure) and the other in response to continuous environments (e.g., physical activity, nutritional measurements, or continuous socioeconomic measures). Here we develop a novel model to jointly account for dichotomous and continuous environments. We develop the model in terms of a joint genotype-by-sex (for the dichotomous environment) and genotype-by-social determinants of health (SDoH; for the continuous environment). Using this model, we show how a depression variable, as measured by the Beck Depression Inventory-II survey instrument, is not only underlain by genetic effects (as has been reported elsewhere) but is also significantly determined by joint G×Sex and G×SDoH interaction effects. This model has numerous applications leading to potentially transformative research on the genetic and environmental determinants underlying complex diseases.

## 1. Introduction

There is presently a robust body of approaches to modeling the genotype-by-environment (G×E) interaction in statistical genetics developed from within a linear mixed model framework, which can be divided into genotype-by-dichotomous environment [1,2,3,4,5,6,7,8] and genotype-by-continuous environment [1,2,7,9,10,11,12,13] interaction models. The former class has been applied to sex (male/female) [1,4,5,6,7], disease affection status (affected/unaffected), or presence/absence of environmental exposures (e.g., smoking/non-smoking, basal/high cholesterol–high-fat diet) [2]. The latter class has been applied to continuous environments such as the age continuum [7,9], physical activity levels and sedentary behavior [10], and socioeconomic status variables [8,13] (e.g., socioeconomic index, household income, or education levels) among others. It would be advantageous to biomedical investigations to combine the two approaches to address interesting questions like a dichotomous sex-specific response to a continuous index of social determinants of health (SDoH), as we do here, or disease affection-status-specific responses to continuous environmental exposures, for example. Deeper reflection shows a plethora of invigorating, potentially transformative research that could be carried out using such a joint interaction model. Here, we review the G × E interaction models for dichotomous and continuous environments and demonstrate for the first time that they can be integrated into a single unified model. The results of our review show that the joint model can uncover important patterns and phenomena that neither model can elucidate alone.

Since the work of Sir Michael Marmot and colleagues beginning in the seventies, SDoH has come to the forefront of biomedical studies aimed at elucidating the causal mechanisms underlying disease [14,15,16,17]. Not surprisingly, the role of SDoH subsequently became an active area of research and application in mental health, particularly depression [18,19,20,21,22,23,24,25,26]. In physiological research, stress in general and SDoH-associated stress have been linked to depression, mainly by the hypothalamus–pituitary–adrenal (HPA) axis [27,28,29,30], which is the primary physiological system that deals with stress [31,32]. We theorize that SDoH may be conceptualized as a complex environment [33] in which the genetic factors underlying depression respond dynamically. That is, there is an observable genotype-by-environment (G × E) interaction between the genetic basis of depression on the one hand and the (composite multivariate) SDoH environment on the other. The SDoH environment may trigger our genetically based stress response system, which may lead to mental health disorders such as depression or anxiety. Moreover, there is an additional layer of complexity to consider because there is now ample evidence of sex-specific genetic effects underlying depression [34,35,36,37]. Thus, it is theoretically possible that the sex and SDoH environments may jointly influence depression outcomes in non-trivial ways. Our novel joint interaction model seeks to investigate how the genetic effects underlying depression may be jointly influenced by the sex and SDoH environments.

## 2. Materials and Methods

The University of Texas Rio Grande Valley IRB approved the study protocol. All participants provided informed consent before participating in the study. We evaluated 522 Mexican American participants recruited from the community in an ongoing genetic study for the presence of obesity, diabetes, hypertension, hyperlipidemia, and depression.

The Beck Depression Inventory II (BDI-II) was used to assess the degree of depressive symptoms over two weeks [12,38]. The BDI-II is a reliable screening tool for assessing the severity of depression, and it can be administered in both Spanish and English. The BDI-II assesses the severity of depression and is an acceptable screening instrument for depression when administered in both Spanish and English [39,40,41].

For our measure of SDoH, we used the survey of health-related social needs screen developed by Billioux et al. (2017)—known as the Accountable Health Communities Health-Related Social Needs (AHC HRSN) screen—comprising ten questions covering housing instability (2 questions), food insecurity (2 questions), transportation needs (1 question), utility needs (1 question), and interpersonal safety (4 questions) [42]. The responses to the ten questions were summed and then divided by 10.

Our main variable of interest in this report is depression as measured by the BDI-II screening instrument. The dichotomous and continuous environments of interest are respectively gender and a derived SDoH environment described in greater detail below.

### 2.1. Statistical Genetic Models and Inference

#### 2.1.1. Polygenic Model

For a generic phenotype vector **y**, assumed to follow a multivariate normal distribution, we write the following:

y=Xβ+g+e

where **X** is a matrix of covariates augmented at the left by a column of 1s, **β** is a vector of the intercept parameter and corresponding regression coefficients, and **g** and **e** are unobserved random genetic and environmental effects, respectively [43,44,45]. The phenotypic covariance matrix, denoted by **Σ**, is given as follows:

$$\Sigma =K\sigma_{g}^{2}+I\sigma_{e}^{2},$$

where **K** and **I**, respectively, denote the genetic relationship and identity matrices, and $\sigma_{g}^{2}$ and $\sigma_{e}^{2}$ are correspondingly the additive genetic and environmental variance components. In statistical genetics, this base model is called the polygenic or additive genetic model [43,44,45,46]. In our initial polygenic models, we accounted for age, sex, age-squared, age-by-sex, and age-squared-by-sex as covariates, and then subjected the residuals to an inverse normalization transformation to induce agreement with the normality assumption [44]. The polygenic model is used to obtain estimates of trait heritability, defined as the ratio of the additive genetic variance to the total phenotypic variance, $h^{2}=\frac{\sigma_{g}^{2}}{\sigma_{g}^{2}+\sigma_{e}^{2}}=\frac{\sigma_{g}^{2}}{\sigma_{p}^{2}}$, and as a model reference point upon which more complex models can be elaborated.

Using the polygenic model, the initial SDoH variable (the AHC HRSN score) was found to have a moderate heritability ($h^{2}$ = 0.47; *p* = 4 × 10^−6^). Given our objective of analyzing SDoH strictly as an environment, we, therefore, employed a best linear unbiased prediction (BLUP) approach to extract the genetic effects that then yielded a predicted value of the environmental effects, which, in turn, can be thought of as a representation of the SDoH variable no longer confounded by genetic effects [45,47]. We term this latter BLUP-derived variable the SDoH Index (SDHI) and use it as our SDoH environmental variable.

#### 2.1.2. Modeling the Genotype-by-Environment Interaction for Discrete and Continuous Environments

For a sample of related individuals, assuming fully uncorrelated genetic and environmental effects, the polygenic model posits that the phenotypic covariance is decomposable into additive genetic and residual environmental variance components and that inter-individual covariances are determined strictly by the additive genetic variance weighted by the genetic relatedness coefficient (see the polygenic covariance equation). The latter feature of the polygenic model makes two implicit assumptions regarding the genetic covariance: that the pairwise genetic correlation is unity and that the additive genetic variance is homogeneous. Explicitly modeling these assumptions is key to our approach to modeling the G×E interaction.

For the simplest case of contrasting two different environments, the G × E variance is zero if the following two conditions are simultaneously true: homogeneity in the additive genetic variance, $\sigma_{g1}^{2}=\sigma_{g2}^{2}=\sigma_{g}^{2}$, where $\sigma_{g1}^{2}$ and $\sigma_{g2}^{2}$ are the additive genetic variances in environments 1 and 2 (for example, male and female status for a G × Sex model or unaffected and affected status for a G × dichotomous disease model), respectively; the genetic correlation ($\rho_{g}$) is one across environments, $\rho_{g}=1$ [1,6,10,12]. Rejection of either or both is evidence that the phenotypic response to the environment has a genetic basis.

We formulate the G × E model for discrete environments in terms of the G × Sex model, with which our group has had great success in elucidating interesting G × Sex or G × dichotomous environment interaction effects (for more detail, see the Appendix A) [4,6]. Under the G × Sex model, the total phenotypic covariance can be decomposed into (1) a within-female polygenic model; (2) a within-male polygenic model; and (3) the across-sex additive genetic covariance. The G × Sex model has parameters $\sigma_{gf}^{2}$, $\sigma_{gm}^{2}$, $\sigma_{ef}^{2}$, $\sigma_{em}^{2}$ for the sex-specific additive genetic and environmental variances and $\rho_{G\left(f,m\right)}$ for the across-sex genetic correlation.

We can extend this theory to an environmental spectrum to model G × E for arbitrary continuous environments as opposed to two levels of the environmental variable (for more detail, see the Appendix A). To this end, we employ variance and correlation functions [10,12,13], which we define as follows:

$$\sigma_{g}^{2}=exp\left[\alpha_{g}+\gamma_{g}\left(q_{i}-\bar{q}\right)\right],\;\mathrm{and}\;\rho_{g}=exp\left(-\lambda_{g}\left|q_{i}-q_{j}\right|\right),$$

where the additive genetic variance is reparameterized as an exponential function of the value of the environmental variable q for the ith individual, $q_{i}$, scaled against the sample mean, $\bar{q}$, and where the genetic correlation is reparameterized as an exponential decay function of the difference of environmental variables for any pair of individuals i and j, and where $\alpha_{g}$, $\gamma_{g}$, and $\lambda_{g}$ are parameters to be estimated. The statistical null hypotheses under the reparameterizations for variance homogeneity and genetic correlation stationarity at unity, respectively, are given as $\gamma_{g}=0$ and $\lambda_{g}=0$. To guard against model misspecification bias, we also model the residual environmental variance as a function of the environment in the same way as the additive genetic variance. Thus, the G × SDHI model has parameters $\alpha_{g}$, $\gamma_{g}$, $\alpha_{e}$, $\gamma_{e}$, and $\lambda_{g}$.

#### 2.1.3. Joint Genotype-by-Environment Interaction for Discrete and Continuous Environments

We now have the elements to construct a model to allow for a joint accounting of the G × E interaction under both a dichotomous environment and a continuous environment (sex and SDHI in the current case, respectively). The full model (detailed in the Appendix A) is decomposed into five equations specifying the within-female variance (1) and (2) covariance, the within-male variance (3) and (4) covariance, and across-sex covariance functions (all functions of the SDHI environment). The joint model has 11 total parameters, namely $\alpha_{gf}$, $\gamma_{gf}$, $\lambda_{gf}$, $\alpha_{ef}$, $\gamma_{ef}$, $\alpha_{gm}$, $\gamma_{gm}$, $\lambda_{gm}$, $\alpha_{em}$, $\gamma_{em}$, and $\rho_{G\left(f,m\right)}$.

### 2.2. Statistical Inferential Theory

For the basic polygenic model, denote the parameter vector by $\theta ={\left[\beta ,\sigma_{g}^{2},\sigma_{e}^{2}\right]}^{\prime }$ and the residuals vector by $\varepsilon =\left(y-X\beta \right)$. On assuming multivariate normality of the phenotype vector, the log-likelihood function is as follows:

$$lnL\left(\theta |y,X\right)=-\frac{1}{2}\left[Nln2\pi +ln\left|\Sigma \right|+\varepsilon^{\prime }\Sigma^{-1}\varepsilon \right]$$

The statistical genetics package SOLAR was used to obtain the model likelihoods, maximum likelihood estimates (MLEs) of model parameters, and their standard errors (SEs) [48]. Hypothesis tests were performed by way of the likelihood ratio test (LRT) statistic, which is given as follows:

$$\Lambda =-2\left[lnL\left(\theta_{N}\right)-lnL\left(\hat{\theta }\right)\right],$$

where for the simplest example, $\theta_{N}$ denotes the parameter vector for which a single parameter is constrained to 0 and all other parameters are free to be estimated at their MLEs, and $\hat{\theta }$ denotes the fully unconstrained parameter vector. In this case, the LRT is distributed as a chi-square random variable with degrees of freedom (df) given by the difference of the number of constrained and unconstrained parameters, which in this simplest case is 1 df: $\Lambda \sim \chi_{1}^{2}$.

It is necessary at this point to distinguish between so-called standard and non-standard conditions. Under standard conditions, the null hypothesis is not on a boundary of the acceptable parameter space, in which case the usual asymptotics for the limiting distribution of the LRT hold. For example, a regression coefficient, being essentially a slope term, takes values on the real line and, more to the point, the null hypothesis of β=0 is not on a boundary. Under non-standard conditions, however, the null hypothesis is on a boundary of the parameter space, in which case the asymptotic limiting distribution for a 1 parameter difference can be shown to be given by a $\frac{1}{2}:\frac{1}{2}$ mixture of a chi-square random variable with a point mass of 0, denoted by $\chi_{0}^{2}$ and $\chi_{1}^{2}$ [44,49,50,51,52,53]. Thus, for this non-standard case, we have $\Lambda \sim \left({\frac{1}{2}\chi }_{0}^{2}+{\frac{1}{2}\chi }_{1}^{2}\right)$. For example, testing $h^{2}=0$ is such a 1-parameter testing scenario where the LRT follows this last mixture distribution.

Our first step is to establish that there is a genetic basis for the trait of interest, which amounts to testing and rejecting $h^{2}=0$. Moving forward from this point, we advocate a staged hypothesis testing approach that our team has successfully developed and implemented in analyses of the gene-by-environment interaction [10,12,13]. In the first stage, we compared the polygenic model to the G × Sex and to the G × SDHI models. Both interaction models are 5-parameter models. Thus, there is an overall 3-parameter difference between the polygenic model (with parameters $\sigma_{g}^{2}$ and $\sigma_{e}^{2}$) and either G × E model. As made clear below in the next stage, 2 of the 3 parameters making up the difference may be considered to be under standard conditions, whereas the remaining parameter can be shown to be under a non-standard condition. Given that, in general, chi-squares are additive, these considerations give rise to $\Lambda \sim \chi_{1}^{2}+\chi_{1}^{2}+\left({\frac{1}{2}\chi }_{0}^{2}+{\frac{1}{2}\chi }_{1}^{2}\right)=\left({\frac{1}{2}\chi }_{2}^{2}+{\frac{1}{2}\chi }_{3}^{2}\right)$.

In the second stage, we examine the specific sources of potential G × E. In particular, we examine the null hypothesis of additive genetic variance homogeneity ($\sigma_{gf}^{2}=\sigma_{gm}^{2}$ and $\gamma_{g}=0$ under G × Sex and G × SDHI, respectively) and a genetic correlation equal to 1 ($\rho_{G\left(f,m\right)}=1$ and $\lambda_{g}=0$ under G × Sex and G × SDHI, respectively). We note that these reparameterizations and their resulting hypothesis tests are predicated on the principle that the likelihood function is invariant under one-to-one reparameterization [46,54,55,56,57,58]. Under the null, the G × Sex model reduces to the polygenic model, whereas the G × SDHI model reduces to a reparameterized polygenic model: $exp\left(\alpha_{g}\right)+exp\left(\alpha_{e}\right)$. The additive genetic variance homogeneity null under the G × Sex model is a standard scenario because it is algebraically equivalent to testing the null hypothesis that their difference equals 0, $\sigma_{gf}^{2}-\sigma_{gm}^{2}=0$, where this difference may take values on the real line and is thus not on a boundary. Regarding the G × SDHI model, $\gamma_{g}$ is essentially a slope term on the log-linear scale and, similar to the case for regression coefficients, the null hypothesis of $\gamma_{g}=0$ is thus not on a boundary. Therefore, for either model, $\Lambda \sim \chi_{1}^{2}$. As for the null hypothesis of a genetic correlation equal to 1 under the G × Sex model, this is clearly on the right boundary of the acceptable parameter space for any correlation coefficient, which takes values in the closed interval $\left[-1,+1\right]$. As for the G × SDHI model, it happens that $\lambda_{g}=0$ is on the left boundary of the permissible parameter space for the exponential decay function, which corresponds to the right boundary of the genetic correlation coefficient because for $\lambda_{g}=0$ we have $\rho_{G}=exp\left(-\lambda_{g}\left|q_{i}-q_{j}\right|\right)=e^{0}=1$. Thus, for both cases, we have $\Lambda \sim \left({\frac{1}{2}\chi }_{0}^{2}+{\frac{1}{2}\chi }_{1}^{2}\right)$.

Under the joint G × Sex and G × SDHI model, we now advocate a third stage, where we test the joint model against whichever of the two 5-parameter models (G × Sex or G × SDHI) has the higher likelihood (this difference need not be significant). The joint model has 11 total parameters, namely $\alpha_{gf}$, $\gamma_{gf}$, $\lambda_{gf}$, $\alpha_{ef}$, $\gamma_{ef}$, $\alpha_{gm}$, $\gamma_{gm}$, $\lambda_{gm}$, $\alpha_{em}$, $\gamma_{em}$, and $\rho_{G\left(f,m\right)}$. Therefore, there is a 6-parameter difference, whereupon focusing on the parameters relevant for G × E testing (or environmental variance heterogeneity), we have 2 parameters on a boundary ($\lambda_{gf}$ and $\lambda_{gm}$) and 4 not on a boundary ($\gamma_{gf}$, $\gamma_{gm}$, $\gamma_{ef}$, and $\gamma_{em}$). The sum of 4 $\chi_{1}^{2}$ variables gives $\chi_{4}^{2}$. Further, to jointly determine the mixture distribution for $\lambda_{gf}$ and $\lambda_{gm}$ we sum their individual mixture distributions as follows: $\left({\frac{1}{2}\chi }_{0}^{2}+{\frac{1}{2}\chi }_{1}^{2}\right)+\left({\frac{1}{2}\chi }_{0}^{2}+{\frac{1}{2}\chi }_{1}^{2}\right)=\left({\frac{1}{4}\chi }_{0}^{2}+{\frac{1}{2}\chi }_{1}^{2}+{\frac{1}{4}\chi }_{2}^{2}\right)$. Therefore, by comparing the joint G × E model to the G × Sex model, we find $\Lambda \sim \chi_{4}^{2}+\left({\frac{1}{4}\chi }_{0}^{2}+{\frac{1}{2}\chi }_{1}^{2}+{\frac{1}{4}\chi }_{2}^{2}\right)=\left({\frac{1}{4}\chi }_{4}^{2}+{\frac{1}{2}\chi }_{5}^{2}+{\frac{1}{4}\chi }_{6}^{2}\right)$.

The fourth stage would then consist of the individual tests with limiting distributions given by their corresponding parameters under either the G × Sex or G × SDHI models. Note that although $\rho_{G\left(f,m\right)}$ was not relevant in deriving the LRT mixture distribution for the comparison of the joint model to the G × Sex model (because this is the only parameter present under both models), it is necessary in this last stage to test if it is significant by using the same procedure given earlier for the G × Sex model.

Before concluding this section, we note that two models, namely the G × SDHI and joint interaction models, had a parameter with a standard error (SE) greater than its corresponding maximum likelihood estimate (MLE). Likelihood theory shows that the MLE of a parameter should be greater than twice its SE if it is significant [57]. Given this principle, we elected to constrain the parameter in question (where the MLE was less than the SE) to its null value following our previously published G × E investigations where this issue was addressed [10,12,13]. To be sure, we also formally tested the parameter by the appropriate test discussed above and confirmed it to be non-significant. In the ensuing sections of this study, such models are referred to as a reduced (abbreviated Red.) version of the model. More importantly, the resulting LRT distributions were accordingly modified by one less parameter following the principles detailed above.

### 2.3. Comparison of Sex-Specific Additive Genetic Variance Functions

In order to enable statistical comparison of the sex-specific additive genetic variance functions at fixed values of the SDHI continuum (minimum, mean, and maximum), we computed their sampling variances using a second-order Taylor expansion approximation for a multivariable non-linear function (see Appendix A) [59].

These sampling variances were then used to compute the adjusted confidence intervals for a two-sided hypothesis test [60]. We note that we have adjusted our confidence interval to correspond to a two-sided test of the hypothesis of no difference [61,62,63]. It can be shown that the proper confidence level corresponding to a significance level of α≤0.05 is conservatively given as 84%, yielding a confidence interval of 8% and 92% for the lower and upper bounds, respectively. We also performed a modified Wald test [46,54,56,57,64] denoted by W, as follows:

$$W=\frac{{\left(\sigma_{gf\left|q\right.}^{2}-\sigma_{gm\left|q\right.}^{2}\right)}^{2}}{\left[Var\left(\sigma_{gf\left|q\right.}^{2}\right)+Var\left(\sigma_{gm\left|q\right.}^{2}\right)\right]}$$

where $\sigma_{gf\left|q\right.}^{2}$ and $\sigma_{gm\left|q\right.}^{2}$ are the sex-specific additive genetic variances for females and males, respectively, expressed as a function of a fixed SDHI value, denoted by q, and where $Var\left(\sigma_{gf\left|q\right.}^{2}\right)$ and $Var\left(\sigma_{gm\left|q\right.}^{2}\right)$ are their respective variance approximations. Given that we are testing if the difference is different from 0, $W\sim \chi_{1}^{2}$.

## 3. Results

In Table 1, we report the demographic characteristics of the study sample by sex. The heritabilities of both BDI-II and AHC HRSN, each estimated under a polygenic model, were both found to be significant (Table 2). In all analyses, the focal variable is BDI-II. Given that we were interested in using the AHC HRSN score as a measure of the SDoH environment, we used BLUP to statistically extract its genetic effects, at which point we termed it the SDoH index (SDHI).

We proceeded to separately test the performance of the G × Sex and G × SDoH models of BDI-II against the polygenic model for this variable. From the formal comparison in Table 3, we infer that the G × Sex model provides a significantly better fit to the data than the polygenic model. Thus, in Table 4, we proceeded to test the individual hypotheses of homogeneity in the additive genetic and residual environmental variances, and of the genetic correlation equal to 1. We found that there is significant G × Sex interaction due to the cross-sex genetic correlation being significantly different from 1. There was also evidence of significant residual variance heterogeneity.

**Figure 1:**
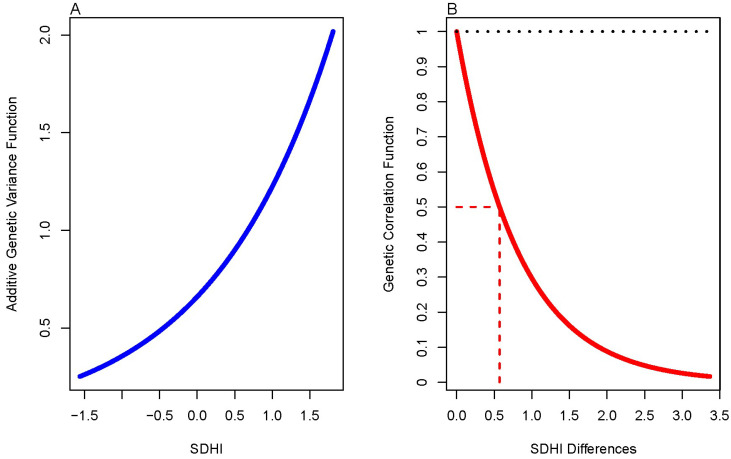
(**A**) Additive genetic variance function. (**B**) Genetic correlation function. The dotted line represents the expected value of the genetic correlation under the null. The red en dash line represents the SDHI value at which the genetic correlation has decayed to half the maximum value.

**Figure 2:**
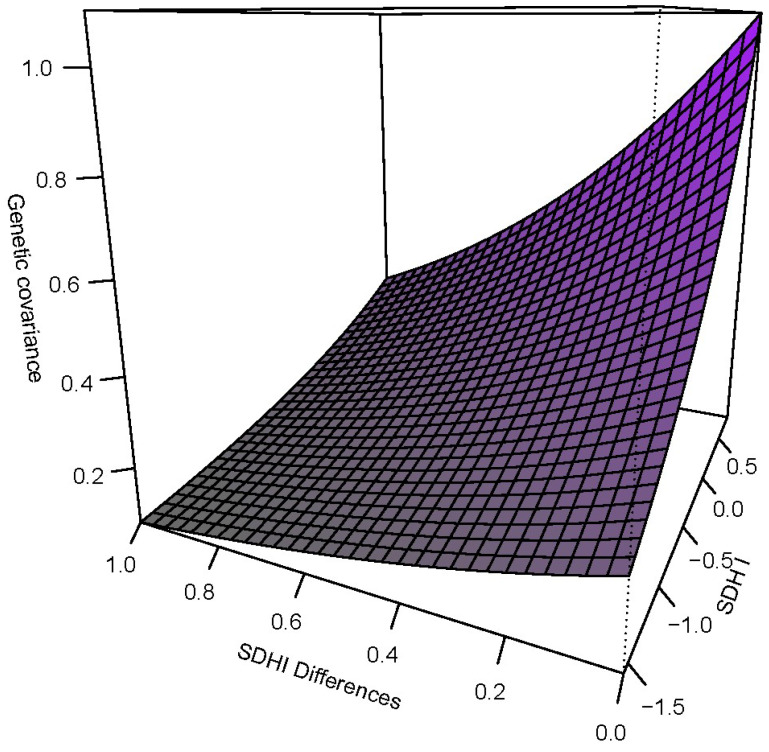
The genetic covariance function showing how the additive genetic variance as a function of SDHI and the genetic correlation as a function of SDHI differences jointly influence the overall covariance.

Similarly, we found that the G × SDoH model performed significantly better than the polygenic model in explaining the data (Table 5). We thus proceeded to test the slopes of the additive genetic and residual environmental variance functions, and whether the genetic correlation function was different from 1 (Table 6). We found that there was evidence of G × SDoH interaction due to both additive genetic variance heterogeneity and a genetic correlation function that decays away from 1 with increasing differences in the environmental index, SDHI (Figure 1 and Figure 2).

Up to this point, we have demonstrated evidence of G × Sex and G × SDoH interactions. However, each interaction analysis was conducted separately from the other, and supposing that our investigation ended here, we could not be certain if both significant interactions hold when considered together. Therefore, we performed a joint interaction model analysis to address whether both interactions are still important when considered together. We selected the G × Sex interaction model to be the baseline model to assess the significance of the joint interaction model because it had a higher likelihood than the G × SDoH interaction model. On comparing the joint interaction model to the G × Sex interaction model, we found that it had a significantly better fit to the data (Table 7). We further investigated the potential significance of the sex-specific variance and correlation functions and the cross-sex genetic correlation (Table 8). We found that there was still evidence of the G × Sex interaction in this case due to the genetic correlation being significantly less than 1. There was evidence of the G × SDoH interaction in males due to additive genetic variance heterogeneity.

**Figure 3:**
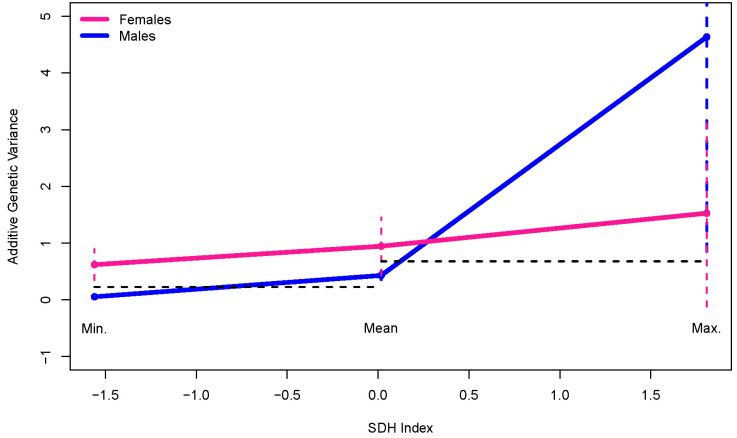
Sex-specific additive genetic variances as functions of SDH Index (SDHI) at the minimum, mean, and maximum SDHI values. The horizontal dashed black lines demonstrate no overlap between the confidence intervals for the male-specific additive genetic variances at the minimum and the mean and at the mean and the maximum. The different color en dash lines indicate the adjusted confidence intervals corresponding to the sex-specific colors (pink for females and blue for males in this case).

Our final analysis compared the sex-specific additive genetic variance functions at the minimum, mean, and maximum SDHI values (Table 9; Figure 3). The male and female additive genetic variance functions significantly differed at the minimum SDHI value but not at the mean or maximum. Consistent with the finding of male-specific additive genetic variance heterogeneity (Table 8), the adjusted confidence intervals for the male-specific additive genetic variances do not show overlap when comparing the variances at the minimum to the mean SDHI values and when comparing the variances at the mean to the maximum SDHI values, indicating a sustained and significant increase in the male-specific additive genetic variance with increasing SDHI (Table 9; Figure 3).

## 4. Conclusions

In this study, we developed a novel model to jointly account for genotype-by-environment interactions for dichotomous and continuous environments. In particular, we applied this joint interaction model to account for the G × Sex and G × SDoH interaction influencing depression. A motivating factor for developing this model is that it allows us to establish if both types of interaction are important independent of one another, similar to the rationale underlying multivariate logistic regression models. We were able to show that there is G × SDoH interaction but only in males and that there is G × Sex interaction due to the cross-sex genetic correlation being significantly different from 1, which indicates that depression is underlain or influenced by different sets of genes in males and females. This model has potentially critical applications in medical research. For example, the dichotomous environment component of the model could be used to model the affected and unaffected states of a given disease of interest, while the continuous environment component could be used to investigate how the genetic response of individuals in both states may be different across a continuous environment of interest, such as physical activity or sedentary behavior. Whatever the case, we are confident that this novel model can lead to fruitful investigations on the genetic basis of response to the environment.

## Figures and Tables

**Table 1:** Demographic characteristics of the sample.

Trait	Females N = 389		Males N = 133		*p*-Value
	Mean	SD	Mean	SD	
Age	44.33	14.76	45.96	15.70	0.2936
BDI-II	19.88	15.99	25.67	19.99	3.1 × 10^−4^
AHA HRSN	0.10	0.14	0.07	0.12	0.0025

**Table 2:** Heritability analysis of BDI-II and AHC HRSN screening on residualized normalized data.

Trait	Heritability	Standard Error	Sample Size	*p*-Value
BDI-II	0.37	0.14	521	7.8 × 10^−6^
AHC HRSN	0.40	0.13	521	6.6 × 10^−4^

**Table 3:** Testing the G × Sex interaction model against the polygenic model for BDI-II.

Trait	Model	Ln Likelihood	Chi-Square	*p*-Value
BDI-II	Polygenic	−247.716	36.97529	2.8 × 10^−8^
	G × Sex	−229.228		

**Table 4:** Testing the critical parameters of the G × Sex interaction model for BDI-II.

Trait	Model	Ln Likelihood	Chi-Square	*p*-Value
BDI-II	Additive genetic variance homogeneity	−229.317	0.178	0.67
	Residual environmental variance homogeneity	−233.465	8.473	1.0 × 10^−3^
	Constrained genetic correlation across sex	−234.850	11.240	4.0 × 10^−4^
	G × Sex interaction model	−229.230		

The LRT statistic for this comparison is distributed as a 50:50 mixture of chi-squares with 2 and 3 degrees of freedom.

**Table 5:** Testing the G × SDHI interaction model against the polygenic model for BDI-II.

Trait	Model	Ln Likelihood	Chi-Square	*p*-Value
BDI-II	Polygenic	−241.401	22.026	9.6 × 10^−6^
	Red. G × E	−230.388		

**Table 6:** Testing the critical parameters of the G × SDHI interaction model for BDI-II.

Trait	Model	Ln Likelihood	Chi-Square	*p*-Value
BDI-II	Constrained genetic slope	−237.646	14.517	1.3 × 10^−4^
	Constrained environmental slope	---	---	---
	Constrained genetic correlation decay	−233.509	6.245	6.2 × 10^−3^
	Red. G × E interaction model	−230.388		

The distributions of the LRT statistics are a chi-square with 1 df and a 50:50 mixture of a chi-square with a point mass at 0 and a chi-square with 1 df. “---” indicates the parameter constrained to the null hypothesis under the reduced G×E interaction model.

**Table 7:** Testing the joint G × Sex and G × SDHI interaction model against the G × Sex for BDI-II.

Trait	Model	Ln Likelihood	Chi-Square	*p*-Value
BDI-II	G × Sex	−229.228	42.73996	1.8 × 10^−8^
	Red. G × E	−207.858		

**Table 8:** Testing the critical parameters of the joint G × Sex and G × SDHI interaction model for BDI-II.

Trait	Model	Ln Likelihood	Chi-Square	*p*-Value
BDI-II	Constrained genetic slope in females	−208.182	0.647	0.42
	Constrained environmental slope in females	−207.942	0.169	0.68
	Constrained genetic correlation decay in females	−208.576	1.436	0.12
	Constrained genetic slope in males	−216.248	16.780	4.2 × 10^−5^
	Constrained environmental slope in males	---	---	---
	Constrained genetic correlation decay in males	−208.28	0.84433	0.18
	Constrained across-sex genetic correlation	−211.642	7.568402	2.0 × 10^−3^
	Red. G × E interaction model	−207.858		

“---” indicates the parameter constrained to the null hypothesis under the reduced G×E interaction model.

**Table 9:** Comparison of sex-specific additive genetic variances at the minimum, mean, and maximum SDHI values (95% confidence interval at the lower and upper bounds).

Sex	Additive Genetic Variance	Adjusted Lower Bound *	Adjusted Upper Bound	Wald Statistic	*p*-Value
Min. SDHI					
Females	0.618	0.347	0.890	6.527	0.011
Males	0.053	0.013	0.092		
Mean SDHI					
Females	0.944	0.437	1.451	1.550	0.213
Males	0.429	0.354	0.503		
Max. SDHI					
Females	1.5253	−0.1185	3.169	0.6485	0.4206
Males	4.6378	0.8509	8.4247		

* See text on the adjusted lower and upper bounds.

## Data Availability

The raw data supporting the conclusions of this article will be made available by the authors on request.

## Appendix A. On the Statistical Genetic Models

### Appendix A.1. Polygenic Model

For a generic phenotype vector **y**, assumed to follow a multivariate normal distribution, we write the following:

y=Xβ+g+e

where **X** is a matrix of covariates augmented at the left by a column of 1s, **β** is a vector of the intercept parameter and corresponding regression coefficients, and **g** and **e** are unobserved random genetic and environmental effects, respectively [43,44,45]. The phenotypic covariance matrix, denoted by **Σ**, is given as follows:

$$\Sigma =K\sigma_{g}^{2}+I\sigma_{e}^{2},$$

where **K** and **I**, respectively, give genetic relationship and identity matrices, and $\sigma_{g}^{2}$ and $\sigma_{e}^{2}$ are correspondingly the additive genetic and environmental variance components. In statistical genetics, this base model is called the polygenic or additive genetic model [43,44,45,46]. It is instructive at this point to provide the following scalar phenotypic covariance equation, which specifies the elements of the covariance matrix for all possible ij-comparisons:

$$\sigma_{p}\left(y_{i},y_{j}\right)=k_{ij}\sigma_{g}^{2}+\delta_{ij}\sigma_{e}^{2}=\left\{\begin{array}{c} \sigma_{g}^{2}+\sigma_{e}^{2}\;\forall \;i=j;k_{ij}=1;\delta_{ij}=1\\ k_{ij}\sigma_{g}^{2}\;\forall \;i\neq j;\delta_{ij}=0 \end{array}\right.$$

where $\sigma_{p}\left(y_{i},y_{j}\right)$ denotes the phenotypic covariance, $k_{ij}$ is the ij-th element in **K**, and $\delta_{ij}=\left\{\begin{array}{c} 1\;\forall \;i=j\\ 0\;\forall \;i\neq j \end{array}\right.$ denotes the Kronecker delta [65,66]. The top case on the right-hand side gives the diagonal elements of the covariance matrix under the polygenic model, and the bottom case gives the off-diagonal elements.

### Appendix A.2. Modeling the Genotype-by-Environment Interaction for Discrete and Continuous Environments

For a sample of related individuals, assuming fully uncorrelated genetic and environmental effects, the polygenic model posits that the phenotypic covariance is decomposable into additive genetic and residual environmental variance components and that inter-individual covariances are given strictly by the additive genetic variance weighted by the genetic relatedness coefficient (see the polygenic covariance equation). The latter feature of the polygenic model makes two implicit assumptions regarding the genetic covariance: that the pairwise genetic correlation is unity and that the additive genetic variance is homogeneous. Explicitly modeling these assumptions is key to our approach to modeling the G × E interaction.

For the simplest case of contrasting two different environments, the G × E variance is zero if the following two conditions are simultaneously true: homogeneity in the additive genetic variance, $\sigma_{g1}^{2}=\sigma_{g2}^{2}=\sigma_{g}^{2}$, where $\sigma_{g1}^{2}$ and $\sigma_{g2}^{2}$ are the additive genetic variances in environments 1 and 2 (for example, male and female status for a G × Sex model or unaffected and affected status for a G × dichotomous disease model), respectively; the genetic correlation ($\rho_{g}$) is one across environments: $\rho_{g}=1$. Denoting the G × E variance as $\sigma_{g\Delta }^{2}$, we have the following expression:

$$\sigma_{g\Delta }^{2}=\left\{\begin{array}{c} \sigma_{g1}^{2}+\sigma_{g2}^{2}-2\rho_{g}\sigma_{g1}\sigma_{g2}\;\forall \;\sigma_{g1}^{2}\neq \sigma_{g2}^{2}\\ 2\sigma_{g}^{2}\left(1-\rho_{g}\right)\;\forall \;\sigma_{g1}^{2}=\sigma_{g2}^{2}=\sigma_{g}^{2} \end{array}\right.$$

There is G × E evidence if either null hypothesis is rejected [1,6,10,12]. Rejection of either or both is evidence that the phenotypic response to the environment has a genetic basis.

We formulate the G × E model for discrete environments in terms of the G × Sex model, with which our group has had great success in elucidating interesting G × Sex or G × dichotomous environment interaction effects [4,6]. Let there be an indicator variable specifying the sex of individuals in the study, denoted by $s_{i}$, as follows:

$$s_{i}=\left\{\begin{array}{c} 1\;\forall \;i\in Z\\ 0\;\forall \;i\notin Z \end{array}\right.$$

where Z is the set of males or, for generic clinical dichotomous variables, individuals in the “1-class” or affected class. The phenotypic covariance can be decomposed under the G × Sex model as follows:

$$\sigma_{p}\left(y_{i},y_{j}\right)=\left\{\begin{array}{c} k_{ij}\sigma_{gf}^{2}+\delta_{ij}\sigma_{ef}^{2}\;\forall {\;s}_{i}=s_{j}=0\\ k_{ij}\sigma_{gm}^{2}+\delta_{ij}\sigma_{em}^{2}\;\forall {\;s}_{i}=s_{j}=1\\ k_{ij}\sigma_{gf}\sigma_{gm}\rho_{G\left(f,m\right)}\;\forall \;\left(s_{i}=1\;and\;s_{j}=0\right)\;or\;\left(s_{i}=0\;and\;s_{j}=1\right) \end{array}\right.$$

Note that the decomposition covers three general classes, which in descending order are the (1) within-female polygenic model; (2) within-male polygenic model; and (3) the across-sex additive genetic covariance. To write the matrix model, let there be a n×1 indicator vector s with elements $s_{i}$, as defined above. To construct incidence matrices for pairwise comparisons consisting of both individuals in the male class and both individuals in the female class, we write, respectively, $M_{n\times n}=s_{nx1}{s^{\prime }}_{1\times n}$ and $F_{n\times n}=r_{nx1}{r^{\prime }}_{1\times n}$, where r=1−s and 1 is a vector of 1s [4]. For the across-class pairwise comparisons, we write $C_{n\times n}=s_{nx1}{r^{\prime }}_{1\times n}+r_{nx1}{s^{\prime }}_{1\times n}$ [4]. The covariance matrix for this model may be given as follows:

$$\Sigma =K⨀\left(F\sigma_{gf}^{2}+M\sigma_{gm}^{2}+C\sigma_{gf}\sigma_{gb}\rho_{G\left(f,m\right)}\right)+I⨀\left(F\sigma_{ef}^{2}+M\sigma_{ef}^{2}\right),$$

where ⨀ denotes the Hadamard matrix multiplication operator.

We can extend this theory to an environmental spectrum to model G × E for arbitrary continuous environments as opposed to two levels of the environmental variable. To this end, we employ variance and correlation functions [10,12,13], which we define as follows:

$$\sigma_{g}^{2}=exp\left[\alpha_{g}+\gamma_{g}\left(q_{i}-\bar{q}\right)\right],\;\mathrm{and}\;\rho_{g}=exp\left(-\lambda_{g}\left|q_{i}-q_{j}\right|\right),$$

where the additive genetic variance is reparameterized as an exponential function of the value of the environmental variable q for the ith individual, $q_{i}$, scaled against the sample mean, $\bar{q}$, and where the genetic correlation is reparameterized as an exponential decay function of the difference of environmental variables for any pair of individuals i and j, and where $\alpha_{g}$, $\gamma_{g}$, and $\lambda_{g}$ are parameters to be estimated. These functions can be interpreted as the variance and correlation functions of a Gaussian stationary stochastic process [8,13,67,68,69,70], where the index variable of the stochastic process is the SDHI environment. The statistical null hypotheses under the reparameterizations for variance homogeneity and genetic correlation stationarity at unity, respectively, are given as $\gamma_{g}=0$ and $\lambda_{g}=0$. To guard against model misspecification bias, we also model the residual environmental variance as a function of the environment in the same way as the additive genetic variance. The phenotypic covariance function, with components given by the variance and covariance functions of a Gaussian stationary stochastic process, can be partitioned as follows:

$$\begin{array}{cc} \sigma_{p}\left(y_{i},y_{j}\right) & =\left\{\begin{array}{c} \sigma_{g}^{2}+\sigma_{e}^{2}\;\forall \;i=j;\;\delta_{ij}=1;\;q_{i}=q_{j}\\ {{\sigma_{i}\sigma }_{j}\rho }_{g}\;\forall \;i\neq j;\;\delta_{ij}=0;{\;q}_{i}\neq q_{j} \end{array}\right.\\ & =\left\{\begin{array}{c} exp\left[\alpha_{g}+\gamma_{g}\left(q_{i}-\bar{q}\right)\right]+exp\left[\alpha_{e}+\gamma_{e}\left(q_{i}-\bar{q}\right)\right]\;\forall \;i=j;\;\delta_{ij}=1;\;q_{i}=q_{j}\\ {\left\{exp\left[\alpha_{g}+\gamma_{g}\left(q_{i}-\bar{q}\right)\right]\right\}}^{\frac{1}{2}}\;{\left\{exp\left[\alpha_{g}+\gamma_{g}\left(q_{j}-\bar{q}\right)\right]\right\}}^{\frac{1}{2}}\;\\ \;\cdots \times exp\left(-\lambda_{g}\left|q_{i}-q_{j}\right|\right)\;\forall \;i\neq j;\;\delta_{ij}=0;{\;q}_{i}\neq q_{j} \end{array}\right.\\ & =\left\{\begin{array}{c} exp\left[\alpha_{g}+\gamma_{g}\left(q_{i}-\bar{q}\right)\right]+exp\left[\alpha_{e}+\gamma_{e}\left(q_{i}-\bar{q}\right)\right]\;\forall \;i=j;\;\delta_{ij}=1;\;q_{i}=q_{j}\\ exp\left[\alpha_{g}+{\frac{1}{2}\gamma }_{g}\left(q_{i}+q_{j}-2\bar{q}\right)-\lambda_{g}\left|q_{i}-q_{j}\right|\right]\;\forall \;i\neq j;\;\delta_{ij}=0;q_{i}\neq q_{j} \end{array}\right. \end{array}$$

We define a genetic covariance matrix $\Psi =\left\{\psi_{ij}\right\}$, with elements given as follows:

$$\psi_{ij}=\left\{\begin{array}{c} exp\left[\alpha_{g}+\gamma_{g}\left(q_{i}-\bar{q}\right)\right]\;\forall \;i=j;\;\delta_{ij}=;q_{i}=q_{j}\\ exp\left[\alpha_{g}+{\frac{1}{2}\gamma }_{g}\left(q_{i}+q_{j}-2\bar{q}\right)-\lambda_{g}\left|q_{i}-q_{j}\right|\right]\;\forall \;i\neq j;\;\delta_{ij}=0;\;q_{i}\neq q_{j} \end{array}\right.$$

We posit a diagonal matrix $\Omega =diag\left\{\omega_{ii}\right\}$ with diagonal elements containing the residual environmental variance function, $\omega_{ii}=exp\left[\alpha_{e}+\gamma_{e}\left(q_{i}-\bar{q}\right)\right]$. The covariance matrix for this G × E model for continuous environments is then given as follows:

Σ=K⨀Ψ+Ω

### Appendix A.3. Joint Genotype-by-Environment Interaction for Discrete and Continuous Environments

We now have the elements to construct a model to allow for a joint accounting of the G × E interaction under both a dichotomous environment and a continuous environment (sex and SDHI in the current case, respectively). A necessary first step is to posit sex-specific variance and correlation functions subscripted by f for females and m for males. Further, we specify the cross-sex genetic covariance function (as opposed to the separate within-sex genetic covariance functions), denoted by Δ, as follows:

$$\Delta ={\left\{exp\left[\alpha_{gf}+{\frac{1}{2}\gamma }_{gf}\left(q_{i}+q_{j}-2\bar{q}\right)-\lambda_{gf}\left|q_{i}-q_{j}\right|\right]\right\}}^{\frac{1}{2}}$$

$$\times {\left\{exp\left[\alpha_{gm}+{\frac{1}{2}\gamma }_{gm}\left(q_{i}+q_{j}-2\bar{q}\right)-\lambda_{gm}\left|q_{i}-q_{j}\right|\right]\right\}}^{\frac{1}{2}}$$

$$=exp\left[\frac{1}{2}\left({\alpha_{gf}+\alpha }_{gm}+\frac{1}{2}\left(\gamma_{gf}+\gamma_{gm}\right)\left(q_{i}+q_{j}-2\bar{q}\right)-\left({\lambda_{gf}+\lambda }_{gm}\right)\left|q_{i}-q_{j}\right|\right)\right]$$

We can write the scalar covariance equation specifying all possible ij-comparisons as follows:

$$\sigma_{p}\left(y_{i},y_{j}\right)=\left\{\begin{array}{c} exp\left[\alpha_{gf}+\gamma_{gf}\left(q_{i}-\bar{q}\right)\right]+exp\left[\alpha_{ef}+\gamma_{ef}\left(q_{i}-\bar{q}\right)\right]\;\forall {\;{i=j;k}_{ij}=1;\delta_{ij}=1;s}_{i}=s_{j}=0\\ k_{ij}exp\left[\alpha_{gf}+{\frac{1}{2}\gamma }_{gf}\left(q_{i}+q_{j}-2\bar{q}\right)-\lambda_{gf}\left|q_{i}-q_{j}\right|\right]\;\forall \;i\neq j;\;\delta_{ij}=0;q_{i}\neq q_{j}\;;{\;s}_{i}=s_{j}=0\\ exp\left[\alpha_{gm}+\gamma_{gm}\left(q_{i}-\bar{q}\right)\right]+exp\left[\alpha_{em}+\gamma_{em}\left(q_{i}-\bar{q}\right)\right]\;\forall {\;{i=j;k}_{ij}=1;\delta_{ij}=1;s}_{i}=s_{j}=1\\ k_{ij}exp\left[\alpha_{gm}+{\frac{1}{2}\gamma }_{gm}\left(q_{i}+q_{j}-2\bar{q}\right)-\lambda_{gm}\left|q_{i}-q_{j}\right|\right]\;\forall \;i\neq j;\;\delta_{ij}=0;q_{i}\neq q_{j}\;;{\;s}_{i}=s_{j}=1\\ k_{ij}\Delta \rho_{G\left(f,m\right)}\;\forall \;i\neq j;\;\delta_{ij}=0;q_{i}\neq q_{j}\;;\left(s_{i}=1\;and\;s_{j}=0\right)\;or\;\left(s_{i}=0\;and\;s_{j}=1\right) \end{array}\right.$$

In descending order, this scalar covariance equation specifies the variance and covariance for the within-female comparisons (the first two cases), the variance and covariance for the within-male comparisons (the next two cases), and the cross-sex covariance. The matrix model, with obvious subscripts for the sex-specific matrices, is given as follows:

$$\Sigma =K⨀\left(F⨀\Psi_{f}+M⨀\Psi_{m}+C{\Delta \rho }_{G\left(f,m\right)}\right)+F\Omega_{f}+M\Omega_{m}$$

Comparison of sex-specific additive genetic variance functions.

In order to enable statistical comparison of the sex-specific additive genetic variance functions at fixed values of the SDHI continuum (minimum, mean, and maximum), we computed their sampling variances using a second-order Taylor expansion approximation for a multivariable non-linear function [59]. The sampling variance approximation, denoted by $D\left[f\left(y\right)\right]$, may be written as follows:

$$D\left[f\left(y\right)\right]\approx a^{\prime }\Sigma_{y}a-\frac{1}{4}{\left[tr\left(A\Sigma_{y}\right)\right]}^{2}$$

where $y={\left[\begin{array}{cc} \alpha_{s} & \gamma_{s} \end{array}\right]}^{\prime }$ (s=f or m), $f\left(y\right)=exp\left[\alpha_{s}+\gamma_{s}\left(q_{i}-\bar{q}\right)\right]$; a is the vector of first partial derivatives evaluated at the maximum, $a=\frac{\partial f\left(y\right)}{\partial y^{\prime }}{|}_{y=\mu }=\left[\begin{array}{c} exp\left[\alpha_{s}+\gamma_{s}\left(q_{i}-\bar{q}\right)\right]\\ \left(q_{i}-\bar{q}\right)exp\left[\alpha_{s}+\gamma_{s}\left(q_{i}-\bar{q}\right)\right] \end{array}\right]$; A is the Hessian matrix of second partial derivatives evaluated at the maximum, $A=\frac{\partial^{2}f\left(y\right)}{\partial y\partial y^{\prime }}{|}_{y=\mu }=\left[\begin{array}{cc} exp\left[\alpha_{s}+\gamma_{s}\left(q_{i}-\bar{q}\right)\right] & \left(q_{i}-\bar{q}\right)exp\left[\alpha_{s}+\gamma_{s}\left(q_{i}-\bar{q}\right)\right]\\ \left(q_{i}-\bar{q}\right)exp\left[\alpha_{s}+\gamma_{s}\left(q_{i}-\bar{q}\right)\right] & {\left(q_{i}-\bar{q}\right)}^{2}exp\left[\alpha_{s}+\gamma_{s}\left(q_{i}-\bar{q}\right)\right] \end{array}\right]$; and where $\Sigma_{y}$ is the asymptotic covariance matrix of the parameters (which can be computed from the output in SOLAR) [59]. The full approximation contains two more terms that were not included in this study due to our focus being on the immediate vicinity of the MLEs.
